# Traumatic right hemi-diaphragmatic injury: delayed diagnosis

**DOI:** 10.1186/s40792-019-0650-5

**Published:** 2019-06-06

**Authors:** Hassan Tavakoli, Jalal Rezaei, Seyed Amir Miratashi Yazdi, Mehdi Abbasi

**Affiliations:** Amir Alam Hospital, Tehran University of Medical Sciences, Tehran, Iran

**Keywords:** Blunt trauma, Diaphragmatic injury, Delayed diagnosis, Right-sided diaphragmatic injury, Intestinal herniation

## Abstract

**Background:**

Traumatic diaphragmatic injury is known to present with the spectrum of symptoms, and most patients would have some symptoms due to abdominal organ herniation. Majority of injuries tend to present on the left hemidiaphragm but right-sided injuries also occur mostly with subtle, delayed presentation due to the buffering effect of the liver.

**Case presentation:**

A 65-year-old male presented to the emergency department with a complaint of nausea and vomiting and reported no bowel movement or passing of flatus for 5 days. Upon further questioning, he recalled that he fell from a tractor while working in his farm 2 months earlier and sustained blunt trauma to his abdomen. Both chest and abdominal X-rays revealed the niveau formation of the small intestine on the right side above the liver and right hemidiaphragm. Further evaluation with CT scan confirmed the presence of a few small intestinal loops behind the liver and also in the chest through a rupture in the right hemidiaphragm. Exploratory laparotomy was performed. Some small intestine loops had gone behind the liver and through 4 cm rupture in the posterior aspect of the diaphragm into the chest. Displaced intestinal loops were relocated and no sign of ischemia or necrosis was observed. The patient was symptom-free within 2 days and he was discharged after 4 days.

**Conclusion:**

Traumatic injuries of the diaphragm are rare, yet underestimated; therefore, they need a high index of suspicion for timely diagnosis and neglected diagnosis may present with a range of symptoms such as herniation months to years later.

## Background

The diaphragmatic injury is uncommon and it could be the consequence of blunt or penetrating thoracoabdominal trauma. It occurs in approximately 5% of blunt trauma patients undergoing laparotomy [1, 2]. The difficulties in diagnosing diaphragmatic rupture and herniation at the first encounter can significantly affect morbidity and mortality. Although diaphragmatic injury can be evident like herniation of abdominal organs on chest imaging, in hemodynamically stable patients who are conservatively managed, the rate of initially neglected diaphragmatic injury is between 12 and 66%, and it could be also missed during laparotomy [3, 4]. In blunt trauma, most ruptures tend to occur on the left hemidiaphragm specifically on the posterolateral side due to structurally weakness of this area as it is the derivative of the pleuroperitoneal membrane. The right diaphragm is congenitally stronger and right-sided injuries may not be apparent on initial evaluation because the liver protects the defect preventing intestinal herniation. Blunt trauma-related left hemidiaphragmatic ruptures have been reported to range from 68.5 to 87% and it is more frequent than the right [5, 6]. Patients may initially present with no symptoms or signs suggesting diaphragmatic injury, but as the time passes, the diaphragmatic rupture tends to grow larger, and herniation of abdominal organs is more likely to occur, particularly on the left side [7]. Patients with the history of thoracoabdominal blunt trauma complaining of abdominal pain, nausea, and vomiting should be assessed for a possible delayed presentation of diaphragmatic injury as a cause of gastrointestinal herniation. Failure to diagnose and repair the diaphragm can result in intestinal strangulation and its fatal consequences [7]. Herein we present a rare case of right hemidiaphragmatic injury which led to intestinal herniation.

## Case presentation

### History and examination

A 65-year-old male presented to the emergency department with a complaint of nausea and vomiting and reported no bowel movement or passing of flatus for 5 days. Upon further questioning, he recalled that he fell from a tractor while working in his farm 2 months earlier and sustained blunt trauma to his abdomen for which his initial evaluation revealed no serious injury except some bruises.

On physical examination, the patient was alert and responsive, his vital signs were stable, and dry mucous membranes were noted. Abdominal examination revealed distended abdomen, increased bowel sounds, and generalized tenderness without rebound tenderness, guarding, or any other significant findings. Fluid resuscitation and nasogastric (NG) tube insertion were initiated for a patient with a suspected diagnosis of intestinal obstruction. His initial lab tests on admission were normal except a mild increase in amylase level (Table 1).

**Table 1:** Lab results on admission

Laboratory test	Result	Normal range
WBC	7500	4000–10,000 (mm^3^)
RBC	5	4.52–5.9 (million/mm^3^)
PLT	185,000	150,000–450,000 (mm^3^)
Hb	14.9	14–18 (g/dl)
Blood sugar	125	50–200 (mg/dl)
Urea	42	18–55 (mg/dl)
Cr	1.2	0.7–1.4 (mg/dl)
AST	29	0–37 (IU/L)
ALT	19	0–41 (IU/L)
Alkaline phosphatase	199	64–306 (IU/L)
Amylase	121	20–100 (IU/L)
Lipase	38	≤ 60 U/I

### Radiological findings

**Fig. 1:**
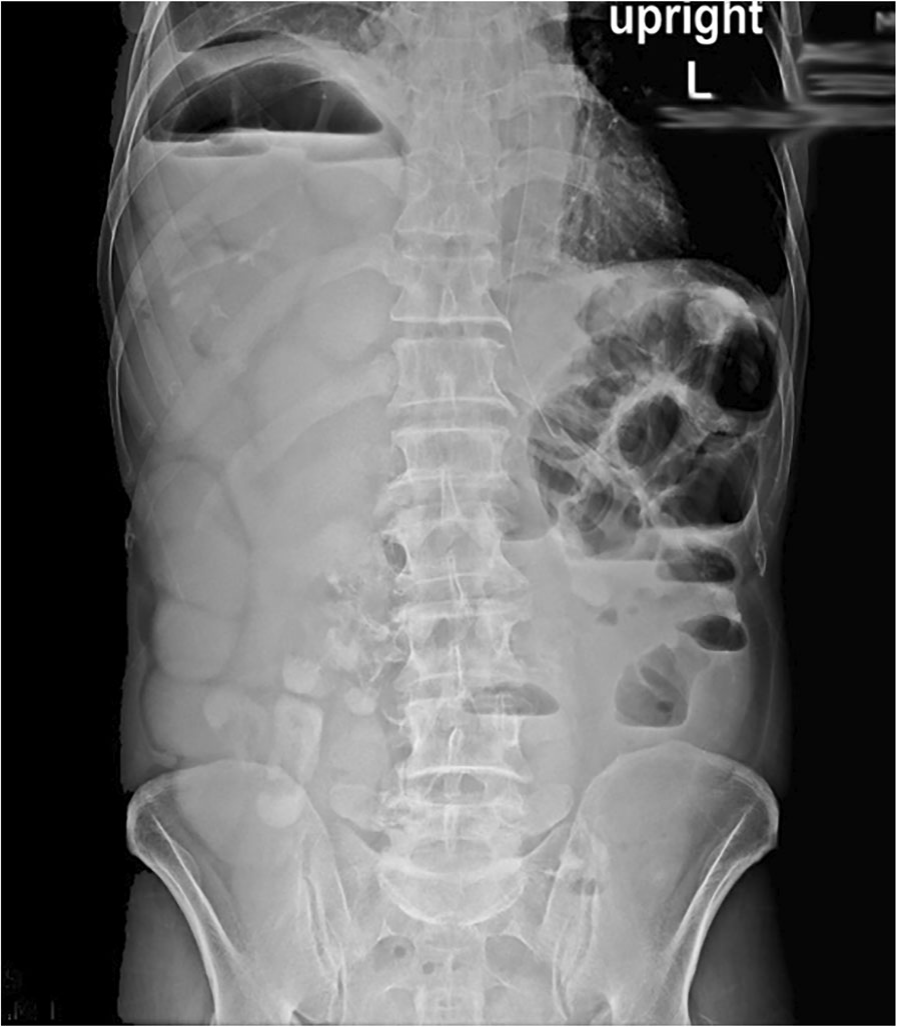
Abdominal X-ray: multiple air-fluid level and air above the liver on the right

**Fig. 2:**
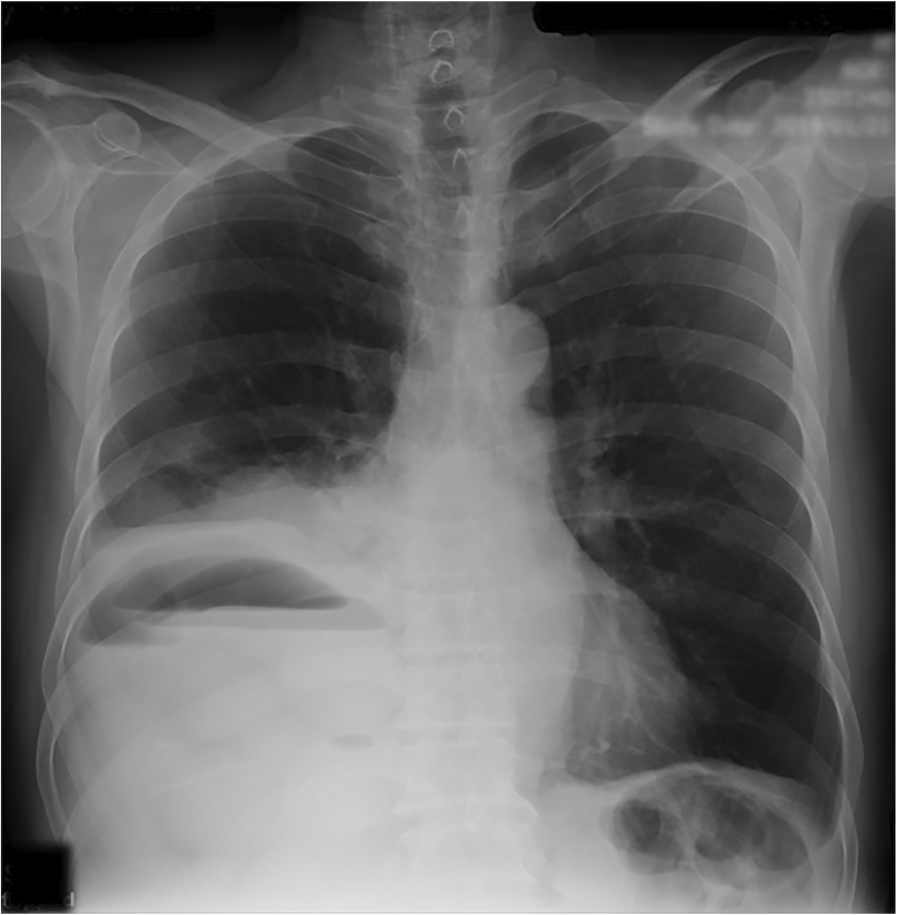
Chest X-ray: air accumulation above the liver

**Fig. 3:**
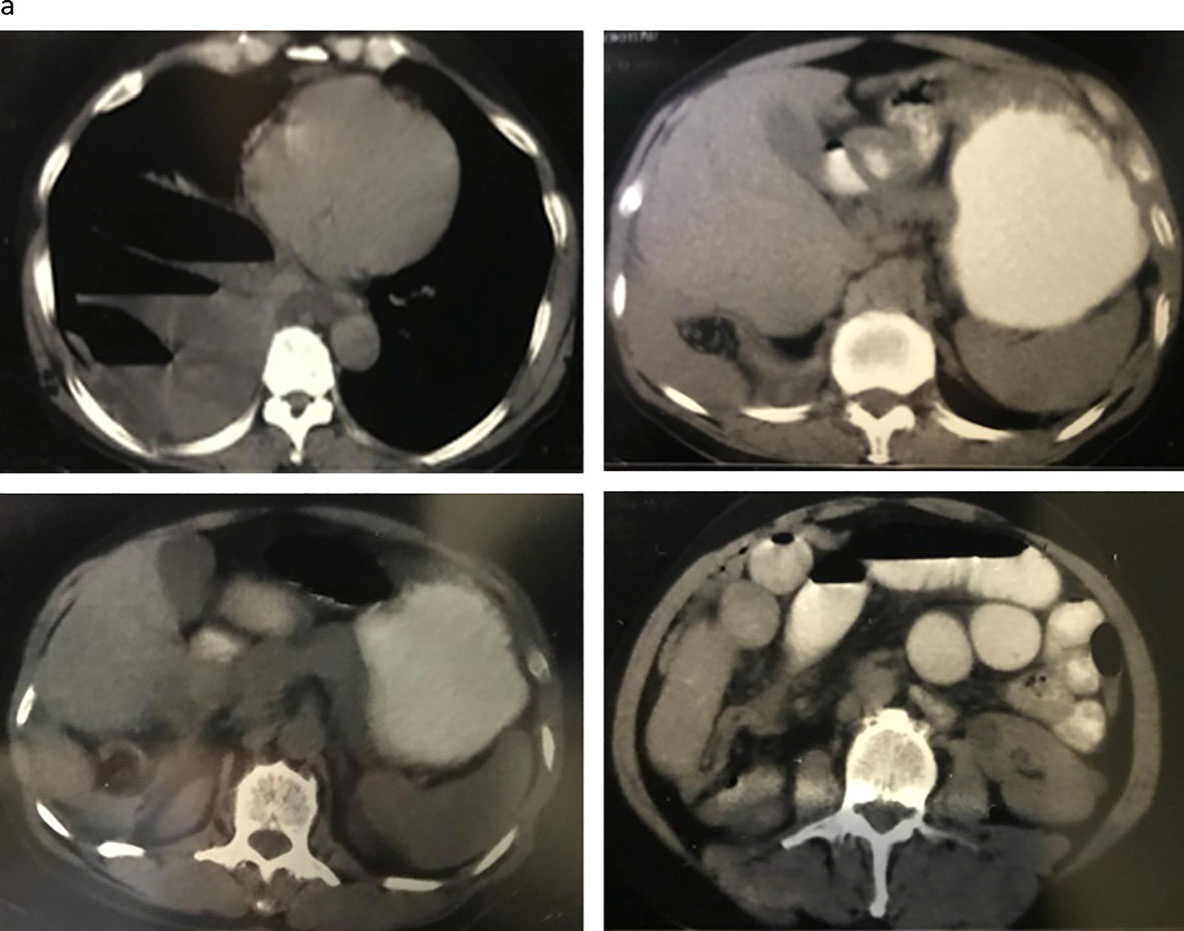
CT scan of patient showing air-fluid level in the intestine, presence of intestinal loop behind the liver through right hemidiaphragm into the right hemithorax

The patient underwent an upright abdominal X-ray and chest X-ray. On the abdominal X-ray, multiple air-fluid levels were observed (Fig. 1). Both chest and abdominal X-rays revealed the niveau formation of the small intestine on the right side above the liver and right hemidiaphragm (Fig. 2). Abdominal sonography reported the presence of dilated intestinal loops. Further evaluation with CT scan confirmed the presence of a few small intestinal loops behind the liver and also in the chest through a rupture in the right hemidiaphragm (Fig. 3).

### Operation

The patient opted for the surgery and exploratory laparotomy was performed. Some small intestine loops had gone behind the liver and through 4 cm rupture in the posterior aspect of the diaphragm into the chest. Displaced intestinal loops were relocated and no sign of ischemia or necrosis was observed. Afterward, the ruptured portion of the diaphragm was closed with Prolene 1 suture by using continuous suturing technique. No other complications were found.

### Postoperative course

The patient had no postoperative complications and he was symptom-free within 2 days. The patient was discharged after 4 days.

## Discussion

Diaphragmatic injuries can present promptly after trauma or have a subtle initial presentation and manifest many months or years later with abdominal organ herniation [7]. Despite advances in imaging studies over the last decade, in the absence of other injuries or organ herniation, diagnosis is difficult due to the complexity of the accompanying injuries and subsequently is usually missed. CT scans can also miss the diagnosis in these injuries [8, 9]. Neglected diaphragmatic injury in the initial presentation can present later with strangulated intestinal diaphragmatic hernia with a high mortality rate [10]. Based on the report by Gelman et al., 66% of patients with a blunt diaphragmatic injury were evaluated insufficiently on initial presentation [11], while Amber et al. reported only 12% of patients with a diaphragmatic injury after blunt trauma were neglected on initial assessment [7]. Isolated diaphragmatic injuries are usually overlooked on initial evaluation unless there is a suspicion of the risk for these types of injuries. In the study by McCune et al., diaphragmatic injuries which were diagnosed on initial presentation had more associated significant organ injuries compared to late presentation settings [7].

Imaging studies can also be inconclusive. In one study, authors concluded that chest X-ray can be inconclusive in up to 59% of patients with diaphragm rupture whereas CT scan can subsequently reveal the defect in these patients [12, 13].

Diaphragmatic injuries still pose a diagnostic challenge to surgeons with a variable spectrum of clinical presentation, and the physiological penalties of these injuries may include gastrointestinal herniation and respiratory symptoms presenting months to years later [14]. Left-sided diaphragm rupture is more likely to occur, and it has been well described in literature while right-sided defect tends to present much lesser due to the protective effect of the liver. To the best of our knowledge, only a few cases of abdominal organ herniation through right-sided diaphragmatic defect have been reported describing the liver as a displaced organ and patients presenting with respiratory symptoms. This case is one of a kind since the patient presented with intestinal obstruction symptoms due to herniation of intestine through a defect in the right hemidiaphragm without liver herniation a couple of months after blunt trauma.

## Conclusion

Traumatic injuries of the diaphragm are rare, yet underestimated; therefore, they need a high index of suspicion for timely diagnosis and neglected diagnosis may present months to years later with a range of symptoms such as herniation. The diaphragmatic injury should be suspected in all blunt thoracoabdominal traumas, and the presence of this injury should be excluded to prevent late complications particularly in stable traumatic patients who could have a subtle presentation.

## Data Availability

Data sharing is not applicable to this article.
